# Production of a Dominant-Negative Fragment Due to G3BP1 Cleavage Contributes to the Disruption of Mitochondria-Associated Protective Stress Granules during CVB3 Infection

**DOI:** 10.1371/journal.pone.0079546

**Published:** 2013-11-18

**Authors:** Gabriel Fung, Chen Seng Ng, Jingchun Zhang, Junyan Shi, Jerry Wong, Paulina Piesik, Lillian Han, Fanny Chu, Julienne Jagdeo, Eric Jan, Takashi Fujita, Honglin Luo

**Affiliations:** 1 Department of Pathology and Laboratory Medicine, University of British Columbia, James Hogg Research Center, Providence Heart+Lung Institute, St. Paul’s Hospital Vancouver, Vancouver, British Columbia, Canada; 2 Laboratory of Molecular Genetics, Institute of Virus Research, Kyoto University, Kyoto, Japan; 3 Laboratory of Molecular Cell Biology, Graduate School of Biostudies, Kyoto University, Kyoto, Japan; 4 Department of Biochemistry and Molecular Biology, University of British Columbia, Vancouver, British Columbia, Canada; University of Hong Kong, Hong Kong

## Abstract

Stress granules (SGs) are dynamic cytosolic aggregates containing messenger ribonucleoproteins and target poly-adenylated (A)-mRNA. A key component of SGs is Ras-GAP SH3 domain binding protein-1 (G3BP1), which in part mediates protein-protein and protein-RNA interactions. SGs are modulated during infection by several viruses, however, the function and significance of this process remains poorly understood. In this study, we investigated the interplay between SGs and Coxsackievirus type B3 (CVB3), a member of the *Picornaviridae* family. Our studies demonstrated that SGs were formed early during CVB3 infection; however, G3BP1-positive SGs were actively disassembled at 5 hrs post-infection, while poly(A)-positive RNA granules persisted. Furthermore, we confirmed G3BP1 cleavage by 3C^pro^ at Q325. We also demonstrated that overexpression of G3BP1-SGs negatively impacted viral replication at the RNA, protein, and viral progeny levels. Using electron microscopy techniques, we showed that G3BP1-positive SGs localized near mitochondrial surfaces. Finally, we provided evidence that the C-terminal cleavage product of G3BP1 inhibited SG formation and promoted CVB3 replication. Taken together, we conclude that CVB3 infection selectively targets G3BP1-SGs by cleaving G3BP1 to produce a dominant-negative fragment that further inhibits G3BP1-SG formation and facilitates viral replication.

## Introduction

In order for Coxsackievirus type B3 (CVB3) to successfully replicate, the virus usurps host protein synthesis machinery. Previous reports on CVB3 infection show cleavage of multiple translation factors, including poly-A binding protein (PABP), eukaryotic translation initiation factor 4 gamma (eIF4G), and eIF5B, by viral protease 2A^pro^ and 3C^pro^ [1]–[3]. Infection by Poliovirus (PV), a closely related enterovirus, also leads to cleavage of multiple translation factors, including PABP, eIF4GI, and eIF4GII [4], [5]. Furthermore, eIF2α phosphorylation upon PV infection results in reduced cytoplasmic concentrations of eIF2-GTP-met-tRNA_i_ ternary complex [6]. Virally-induced shutdown of cap-dependent mRNA protein translation due to cleavage of essential translation factors and phosphorylation of eIF2α results in two outcomes: release of ribosomes for viral protein synthesis and a diminished host antiviral response.

Inhibition of protein synthesis in response to environmental stresses such as oxidative stress, heat shock, and viral infection results in the formation of dynamic cytosolic RNA granules called stress granules (SGs) [7]. SGs comprise, in part, of stalled 48S pre-initiation complexes, including eIF2α, eIF4G, eIF4E, eIF4A, eIF3, 40S subunits, and PABP [8]–[12]. Phosphorylation of eIF2α and inhibition of eIF4A RNA helicase are key effectors that can trigger and lead to the formation of stalled pre-initiation translational complexes and SGs [13], [14]. However, recent studies by Reineke *et al* [15] suggest that large G3BP1-induced granules may precede protein kinase R activation and eIF2α phosphorylation. Under specific situations, SG formation can be uncoupled from translational inhibition [16], suggesting that SG formation only occurs within a fine window of the translation initiation process.

In addition to stalled initiation factors, SGs are composed of more than 50 proteins. A key component of SGs is the aggregating factor, Ras-GTPase-activating protein (GAP) SH3-domain binding protein-1 (G3BP1). G3BP1 is an evolutionarily conserved RNA-binding protein that interacts with Ras-GAP, and is used as a well-established SG marker under a wide range of stresses [17]. Other key proteins that localize to SGs include the T-cell restricted intracellular antigen 1/related (TIA1/R) and HuR [7], [8], [18]. Several studies have reported that SGs may be compositionally different depending on the type of environmental stresses [19], suggesting that distinct SGs may be regulated differentially and have multiple roles. Currently, SGs are thought to be sites of translation-stalled machinery functioning to temporarily store target mRNA, perhaps as an intermediate in processing body formation, an RNA granule involved in the degradation of terminal-mRNA [20]. Furthermore, studies have reported that SGs sequester key factors including receptor for activated C kinase 1 [21] and tumor necrosis factor (TNF) receptor associated factor 2 [22], indicating that SGs may be critical for balancing apoptotic and inflammatory responses, respectively.

It has been shown that SG formation can be modulated by infection of several types of viruses, including flaviviruses, dicistroviruses and picornaviruses. SGs are implicated as both pro- and anti-virus in different infections where multiple viruses have been demonstrated to interfere with different steps of SG assembly [23]. For example, anti-viral SGs are formed early in PV infection, then disassembled at late stages of infection by cleavage of G3BP1 to promote viral replication [24]. Moreover, studies in junin virus demonstrate selective inhibition of anti-viral SG formation by inhibiting eIF2α phosphorylation [25]. Recent studies showed that anti-viral SGs may require mitochondrial-surface proteins to activate downstream interferon induction [26], [27]. These SGs contain anti-viral proteins including retinoic acid-inducible gene 1 (RIG-I), a protein involved in sensing double-strand RNA (dsRNA) for signaling to downstream anti-viral responses by binding interferon-beta promoter stimulator-1 (IPS-1 or also known as MAVS/VISA/Cardif), a mitochondrial surface protein [26], [27]. On the other hand, respiratory syncytial virus and hepatitis C virus has been observed to manipulate SGs for its own benefit by interacting with pro-viral SGs during infection and hijacking critical components, respectively [28], [29].

In this study, we investigated the relationship between CVB3 and SG formation. We demonstrated that CVB3 infection induces the formation of G3BP1-SGs up to 3 hrs post-infection (pi), but active disassembly of G3BP1-SGs occurs beyond 5 hrs pi. Mechanistically, we found that G3BP1-SGs are disassembled via the cleavage of G3BP1 at amino acid Q325 by CVB3 3C^pro^. Furthermore, we observed the protective effects of G3BP1-SGs during CVB3 infection and showed the localization of G3BP1(+)-aggregates near mitochondrial surfaces in CVB3-infected cells. Lastly, we demonstrated that the C-terminal cleavage product of G3BP1 further inhibits G3BP1-SG formation and promotes viral replication. We conclude that cleavage of G3BP1 by 3C^pro^ produces a dominant-negative cleavage fragment that further disrupts mitochondria-associated protective SGs and facilitates viral replication.

## Materials and Methods

### Cell Culture and Materials

HeLa cells were purchased from American Type Culture Collection and grown in complete Dulbecco’s modified Eagle’s medium (DMEM) supplemented with 10% heat-inactivated newborn calf serum.

A monoclonal anti-β-actin antibody was obtained from Sigma. A monoclonal anti-VP1 antibody was purchased from DakoCytomation. Small interfering RNA (siRNA) for G3BP1, siRNA control, horseradish peroxidase-conjugated secondary antibodies, anti-FLAG (D-8, sc-807), anti-TIA-1/TIAR (H-120, sc-28237), anti-eIF4G (D-20, sc-9602), and anti-G3BP1 (H-94, sc-98561) antibodies were purchased from Santa Cruz Biotechnology. Alexa-Fluor-594 goat anti-rabbit IgG and Alexa-Fluor-488 goat anti-mouse IgG were from Molecular Probes. eIF2α (#9722) and phospho-eIF2α (#9721) were purchased from Cell Signaling. Rabbit anti-human PABP antibody was raised against a synthetic peptide sequence (GIDDERLRKEFSPFGTC) in RRM4 of PABP as previous described [30].

### Plasmid Constructs and Transfection

Plasmid encoding GFP-G3BP1 was a generous gift from Dr. Jamal T. at the Institut de Génétique Moléculaire de Montpellier, France. Plasmids of the viral protease 2A driven by the IRES promoter were designed by cloning the open reading frame (ORF) of 2A into the BamHI and SalI sites of the pIRES vector, while 3C-ORF was cloned into the XbaI and SalI restriction sites. N-terminus of G3BP1 (amino acid 1–325) and C-terminus of G3BP1 (amino acid 326–466) were cloned into the p3×FLAG-CMV vector. Transient transfection was conducted as previously described [31]. Briefly, cells grown at a confluence of ∼90% were transiently transfected with plasmids using Lipofectamine 2000 (Life Technologies). siRNA transfections were performed on HeLa cells at 30–50% confluence using Oligofectamine (Life Technologies).

### Virus Infection

HeLa cells were incubated with CVB3 (Kandolf strain) at multiplicities of infection (MOI) of 1 or 10 for 1 hr in serum-free DMEM. The media containing virus were then replaced with fresh complete media until indicated time points. Sham-infected cells were treated with equal volumes of phosphate-buffered saline (PBS).

### Western Blot Analysis

After various time points of infection, the cells were harvested with lysis buffer (250 mM NaCl, pH 7.2, 50 mM Tris-HCl, 0.1% NP-40, 2 mM EDTA, and 10% glycerol). Equal amounts of proteins were loaded for sodium dodecyl sulfate-polyacrylamide gel electrophoresis (SDS-PAGE). Membranes were processed using standard techniques as previously described [31], and immunoreactive bands were visualized by enhanced chemiluminescence.

### Plaque Assay

CVB3 replication was determined by measuring the virus titer in the cell supernatant using an agar overlay as previously mentioned [31]. In brief, supernatant of CVB3-infected cells was serially diluted by 10 fold and overlaid on a 90–95% confluent monolayer of HeLa cells for 1 hr. After PBS wash, cells were overlaid with complete medium containing 0.75% agar and incubated at 37°C for 72 hrs. Carnoy’s fixative (75% ethanol-25% acetic acid) and 1% crystal violet were used for fixation and staining, respectively. Plaques were quantitated and viral titers were calculated based on plaque forming units (PFU) per milliliter.

### Real-time Quantitative RT-PCR

Total RNA was isolated using the RNeasy Mini kit (Qiagen). cDNA was synthesized using SuperScript III First-Strand Synthesis SuperMix (Life Technologies). Primers previously designed to target the 5′ UTR of CVB3 were optimized for SYBR Green quantitative PCR [32]. The 25 µl qPCR reaction mix consisted of 1 nL cDNA template, 500 nM forward and reverse primers, and 1×QuantiTect SYBR Green PCR Master Mix (Qiagen). The PCR program, run on a ViiA 7 Real-Time PCR System (Applied Biosystems), included a 15 min polymerase activation step at 95°C, and 40 cycles of 94°C/15 sec, 53°C/30 sec, and 72°C/30 sec where endpoint fluorescence was recorded. A terminal dissociation curve was included to assess primer specificity and dimer formation. Triplicate samples were run with a 10×serial dilution of sample cDNA as a standard for determining PCR efficiency.

### *In situ* Hybridization

*In situ* hybridization was performed using a poly(A) probe [oligo(dT)40 conjugated to Cy-3 (Sigma)], or antisense-strand CVB3 RNA probe [AAGCCAATCTAAATTATTTCAAATT conjugated to Cy-5 (Sigma)] as previously described [33].

### Indirect Immunocytochemical Microscopy

Indirect immunofluorescence assay was performed as previously described [33]. Following permeabilization, the following primary antibodies were used: 1∶200 rabbit-anti-G3BP1, 1∶200 mouse-anti-FLAG. After washing, the cells were incubated with the following secondary antibodies: 1∶200 Alexa-Fluor-594 goat anti-rabbit IgG (Molecular Probes), 1∶200 Alexa-Fluor-488 goat anti-mouse IgG, 1∶200 Alexa-Fluor-594 goat anti-mouse IgG. Cell nuclei were counterstained with 4′, 6-diamidino-2-phenylindole (DAPI). Images were captured under a Leica SP2 AOBS confocal microscope. The quantification of stress granule formation was performed by counting the number of cells expressing 3 or more punctates and dividing by the number of cells expressing the corresponding fluorophore, where each image should consist of 20 or more cells.

### Transmission Electron Microscopy (TEM)

For visualizing the ultrastructure of SGs, TEM was performed using standard techniques as previously described [34]. In brief, after CVB3 infection, HeLa cells were processed and embedded in Eponate 12 resin (Ted Pella, Inc.), sectioned at a thickness of 60 nm, and viewed on a Tecnai 12 transmission electron microscope (FEI, Inc.).

### Immuno-electron Microscopy (IEM)

IEM processing was performed as previously described [35]. For immune-labeling of G3BP1, rabbit anti-G3BP1 polyclonal antibody (Cat.ab39533, Abcam) was diluted at 1∶60. F(ab’) 2 fragment of ultra-small goat-anti-rabbit IgG was diluted at 1∶50. Following steps were performed using a Pelco Biowave Microwave. Free aldehydes were blocked using 50 mM glycine, blocked in 5% goat serum containing 0.18% cold water fish skin gelatin, washed in acetylated-BSA (BSA-c) and incubated with primary antibody. Controls were incubated in normal goat serum diluted at 1∶60. Sections were washed, and incubated in secondary antibody, sequentially washed in BSA-c, PBS, 2% glutaraldehyde, distilled water, and Silver R-Gent SE-EM. Finally, sections were stained in 2% uranyl acetate, lead citrate, air dried and analyzed on a Tecnai 12 electron microscope.

### Viral Protease Purification

CVB3 2A^pro^ was purified from pET-C×2A expressed in BL21 bacterial cells by ion exchange chromatography and size exclusion chromatography as previously described [36], [37]. CVB3 3C^pro^ was cloned into a pET29b plasmid containing a His-tag and expressed in BL21 bacterial cells for 4 hrs at 30°C. Bacterial cells were lysed by sonication in 50 mM NaCl, 50 mM Tris pH 8, 5 mM Imidazole, 5 mM β-mercaptoethanol, 5% glycerol, then purified by Ni-nitrilotriacetic acid chelating resin affinity chromatography.

### *In vitro* Cleavage Assay

Cleavage reactions were performed in 20 mM Hepes (pH 7.4), 150 mM KOAc and 1 mM DTT. Each sample comprised of HeLa lysates with purified CVB3 2A^pro^ or 2A^pro^ catalytically inactive mutant at 5 ng/µl or CVB3 3C^pro^ at 100 ng/µl. Reaction mixtures were incubated at 37°C for the amount of time as indicated and the reaction was stopped by the addition of SDS-PAGE sample buffer. Cleavage activity was assessed by western blotting.

## Results

### CVB3 Infection Induces SG Formation at ∼3 hrs and Disassembly at ∼5 hrs Post-infection

**Figure 1:**
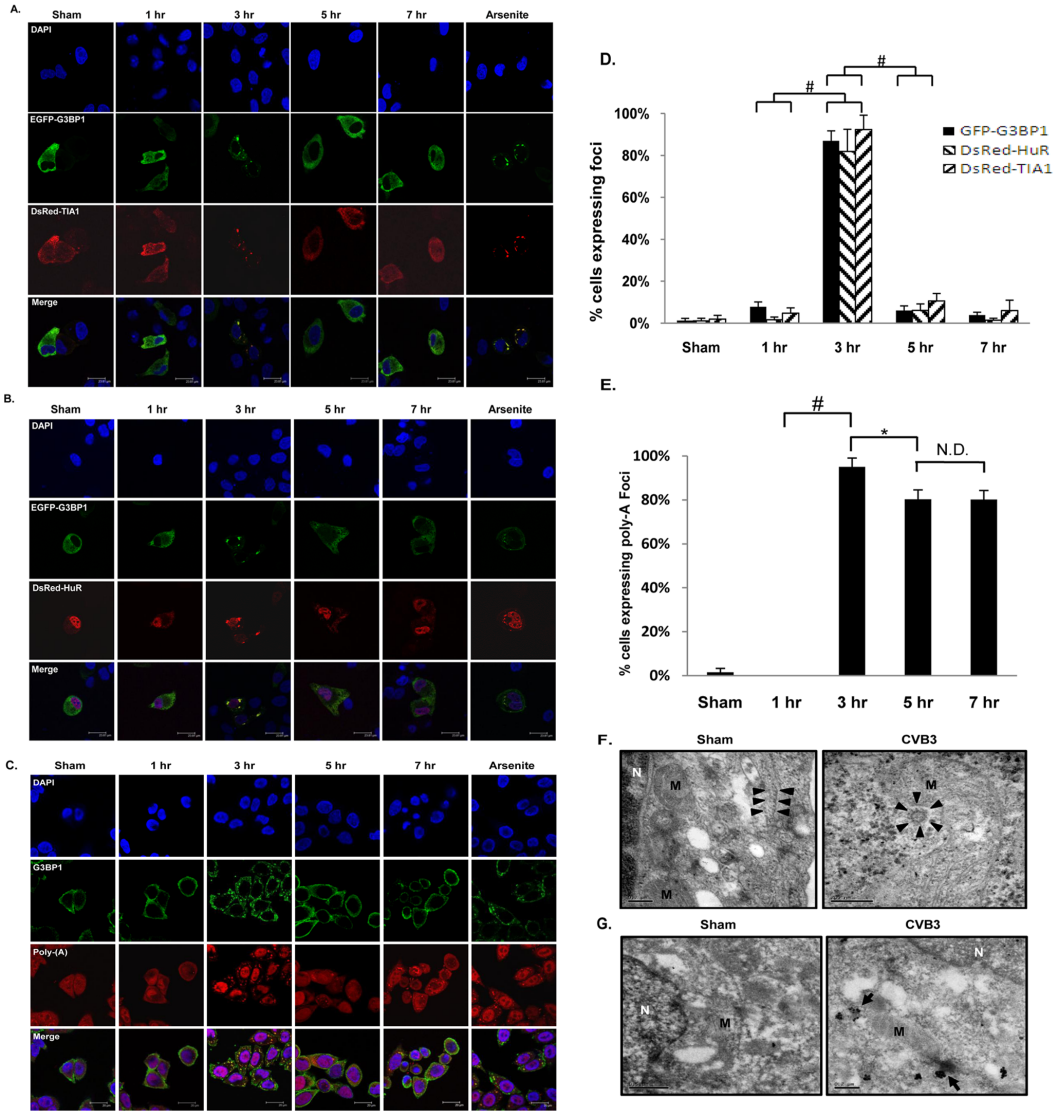
CVB3 infection induces SG formation at ∼3 hrs and disassembly at ∼5 hrs post-infection. (A, B) Representative confocal images of intracellular localization of G3BP1, TIA1 and HuR following CVB3 infection of HeLa cells. HeLa cells were transiently co-transfected with pEGFP-G3BP1 and either pDsRed-TIA1 (A) or pDsRed-HuR (B) for 48 hrs, followed by sham- or CVB3 infection at an MOI of 10 for various time points as indicated. Arsenite treatment at a dose of 50 mM for 1 hr was used as positive controls for inducing SG formation. Cell nuclei were counterstained with 4′, 6-diamidino-2-phenylindole (DAPI). (C) Representative confocal images of co-localization of poly-A-mRNA with G3BP1 during CVB3 infection. HeLa cells were transiently transfected with pEGFP-G3BP1 for 48 hrs, followed by sham- or CVB3 infection at an MOI of 10 for different time points as indicated. Poly-A-mRNA was detected by *in situ* hybridization using an oligo-dT probe synthetically conjugated to Cy3, followed by immunostaining for G3BP1. Cell nuclei were counterstained with DAPI. (D) Quantitation of G3BP1-SG formation from (A, B). Percent of cells expressing SGs was quantified as described in Materials and Methods (mean ± SD, n = 10 images), #p<0.001. (E) Quantitation of poly-A granules from (C). Percent of cells expressing poly-A punctates was quantified as above. N.D., No statistical difference, *p<0.01. (F, G) Representative transmission (F) and immune-electron (G) microscope images. HeLa cells were either sham- or CVB3-infected at an MOI of 10 for 4 hrs. N and M indicate the nucleus and mitochondria, respectively. Arrow heads in (F) indicate ribosomal-like structures. Arrows in (G) indicate cytoplasmic aggregates that were stained positive for endogenous G3BP1.

Regulation and function of SG formation have been implicated in other viruses including PV and Encephalomyocarditis virus (EMCV) in the *Picornaviridae* family [24], [27]; however the effect of CVB3 infection on SG formation has not been investigated. In order to visualize SG formation in CVB3 infection, we utilized HeLa cells stably expressing green fluorescent tagged G3BP1 (GFP-G3BP1), a protein involved in the assembly of SGs. Confocal imaging showed the punctate accumulation of GFP fluorescence in the cytosol at ∼3 hrs post-infection (pi) and disappearance at ∼5 hrs pi (**Figure 1A, B, and D**). Using two other well established SG markers, TIA1 and HuR, we demonstrated co-localization of red punctates (dsRed-TIA1 or HuR) with green punctates (GFP-G3BP1) (**Figure 1A, B, and D**), further suggesting formation and disassembly of SGs during CVB3 infection. Live-cell fluorescent imaging using GFP-G3BP1 stable cell lines showed similar results (**Figure S1**). Furthermore, we examined whether G3BP1-SGs co-localize with poly(A)-mRNA. We demonstrated the co-localization of G3BP1-SGs with poly(A)-mRNA at 3 hrs pi; however at 5 hrs pi, G3BP1 dissolved homogenously back into the cytoplasm while poly(A)-positive granules persisted (**Figure 1C and E**). The persistence of RNA granules suggests that G3BP1-SGs are specifically targeted during CVB3 infection. Our preliminary results have suggested that the formation and persistence of poly(A)-granules observed at 5 hrs and 7 hrs pi are associated with other RNA granules, i.e. P-bodies (data not shown).

To gain insight into the cellular localization of stalled initiation complexes in CVB3-infected cells, we performed transmission- and immuno-electron microscopy analyses. We chose 4 hrs as an endpoint in this study because SGs are still present at high levels before disassembly at 5 hrs pi. As shown in **Figure 1F**, in sham-infected cells, the ribosomal-like particles (indicated by arrow heads) were mainly localized to the fiber-like structures that represent the endoplasmic reticulum. However, in CVB3-infected cells, these particles tended to accumulate to form aggregates (∼100 nm in diameter, indicated by arrow heads), similar to those shown by Gilks *et al.* [34]. Furthermore, immune-electron microscopy imaging showed that G3BP1 staining was diffuse in sham-infected cells, while CVB3-infected cells comprised of both diffuse and G3BP1-positive aggregates, the latter were primarily observed near mitochondrial surface (**Figure 1G**). This observation further supports the presence of G3BP1-positive aggregates in CVB3-infected cells.

### Coxsackieviral Protease 2A Cleaves eIF4G and Promotes SG Formation

**Figure 2:**
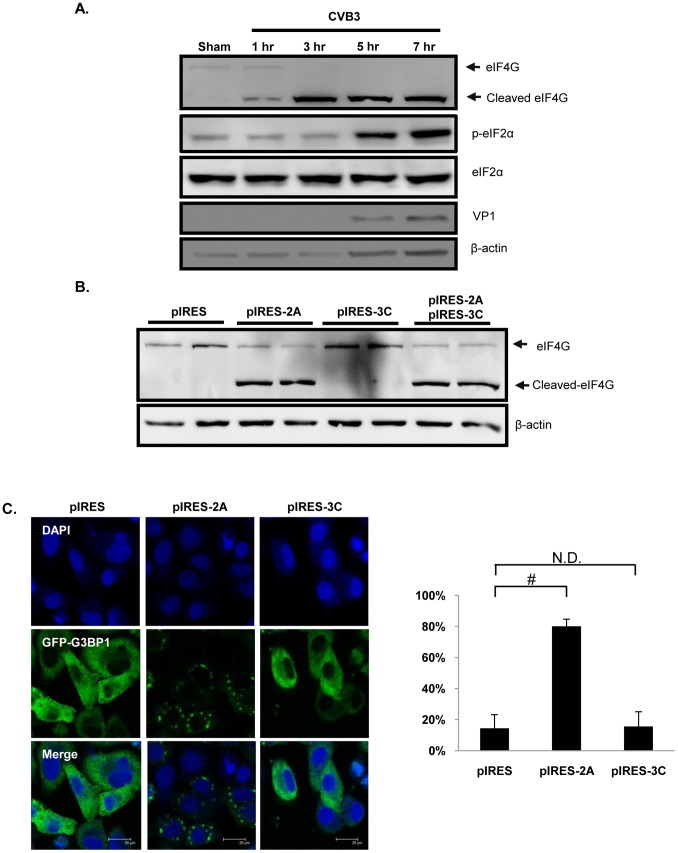
Coxsackieviral protease 2A cleaves eIF4G and promotes SG formation. (A) HeLa cells were either sham- or CVB3-infected at an MOI of 10 for various time points as indicated. Western blotting was performed to assess protein expression of eIF4G, phopho-eIF2α (Ser 51), and total eIF2α. Protein level of viral capsid protein (VP1) and β-actin was also examined as an infection and loading control, respectively. (B) HeLa cells were transfected with pIRES-2A and/or pIRES-3C for 48 hrs. Western blotting was performed to assess protein expression of eIF4G and β-actin. (C) Representative confocal images of viral protease 2A^pro^ inducing G3BP1-SG formation. HeLa cells were co-transfected with pIRES-2A^pro^ or pIRES-3C^pro^ together with GFP-G3BP1 for 24hrs, followed by confocal microscopy analysis.

It has been previously demonstrated that CVB3 infection inhibits cap-dependent protein translation, mainly due to eIF4G cleavage and eIF2α phosphorylation [1], [31]. To explore the potential mechanism by which CVB3 regulates SG formation, we examined the kinetics of eIF4G cleavage and eIF2α phosphorylation. We found that eIF4G cleavage occurred at ∼1 hr pi preceding eIF2α phosphorylation at ∼5 hrs pi (**Figure 2A**). It should be noted that the un-correlation of band intensities between full-length and cleaved eIF4G in Figure 2A is likely a result of incomplete transfer of high molecular weight protein (i.e. full-length eIF4G ∼220kDa). Using IRES-driven CVB3 2A^pro^ and 3C^pro^ constructs, we showed that eIF4G was cleaved in HeLa cells transfected with pIRES-2A^pro^ (**Figure 2B**). Furthermore, we demonstrated that cells expressing pIRES-2A^pro^, but not pIRES-3C^pro^, displayed increased G3BP1 foci (**Figure 2C**). Together, these results suggest that translation initiation inhibition, which is in part due to eIF4G cleavage by 2A^pro^, leads to enhanced SG formation during the early phase of CVB3 infection.

### CVB3 Infection Causes G3BP1 Cleavage

**Figure 3:**
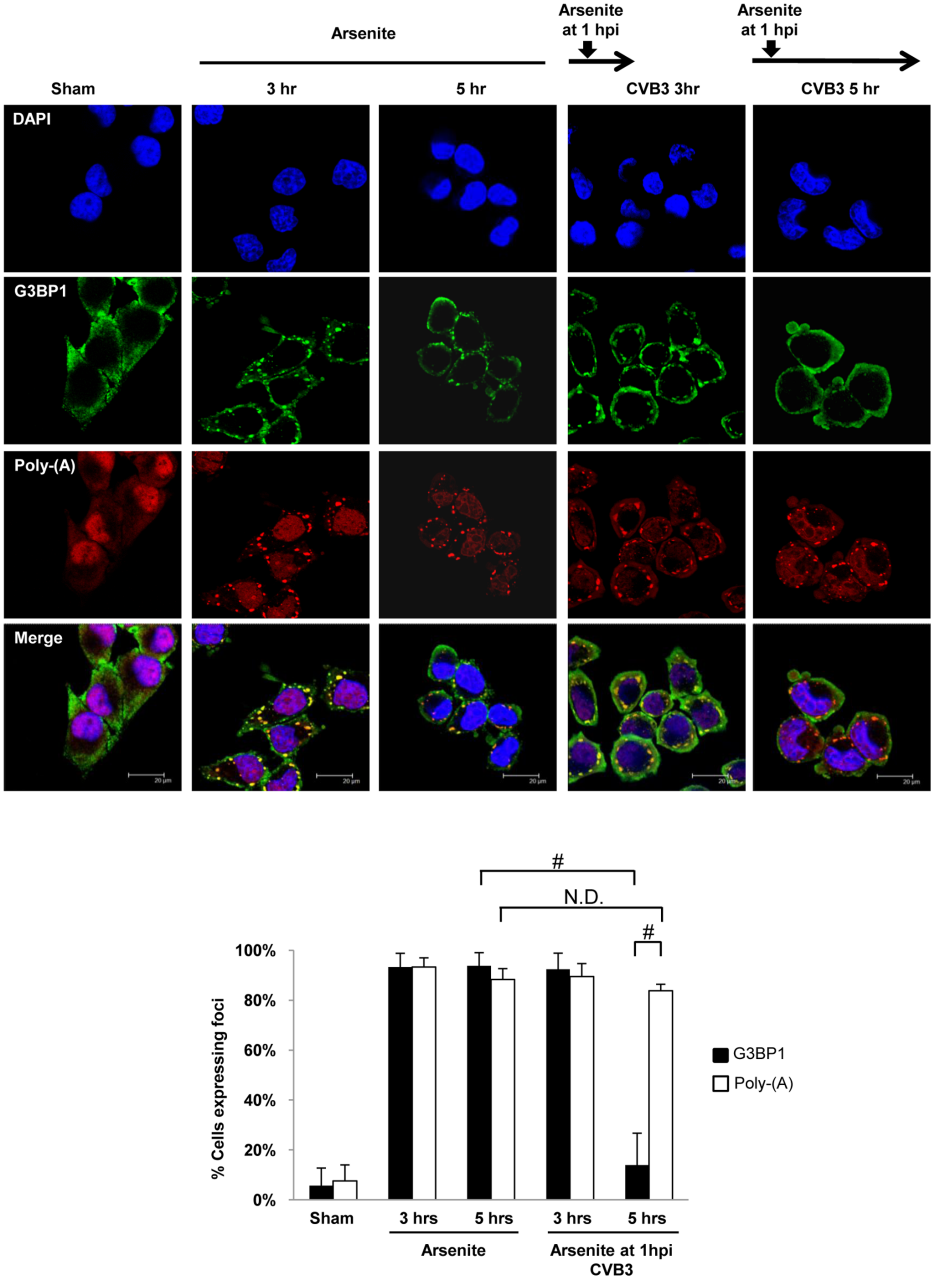
G3BP1-SGs are disassembled while poly-A-SGs remain persistent in CVB3-infected cells. HeLa cells were infected with CVB3 at an MOI of 10 for 3 or 5-infection (pi) as indicated. Cells treated with arsenite alone for 3 or 5 hrs were used as negative controls. Cells were fixed and stained for endogenous G3BP1, poly-A mRNA, and nuclei. Percent of cells expressing G3BP1-SGs and poly-A granules was quantified as described in Materials and Methods (mean ± SD, n = 5 images), N.D., No statistical difference, #p<0.001.

To investigate whether disappearance of SGs at the late stage of CVB3 infection is due to an inhibition of the assembly of SGs and/or a result of increased disassembly of SGs, HeLa cells infected with CVB3 were treated with arsenite to induce SG formation and G3BP1-SGs were monitored at 3 hrs and 5 hrs pi. **Figure 3** showed that arsenite-induced G3BP1-SGs were significantly inhibited after 5 hr CVB3 infection, whereas poly-A-SGs persisted. This data suggests that G3BP1-SGs are actively and selectively disassembled beyond 5 hrs pi.

**Figure 4:**
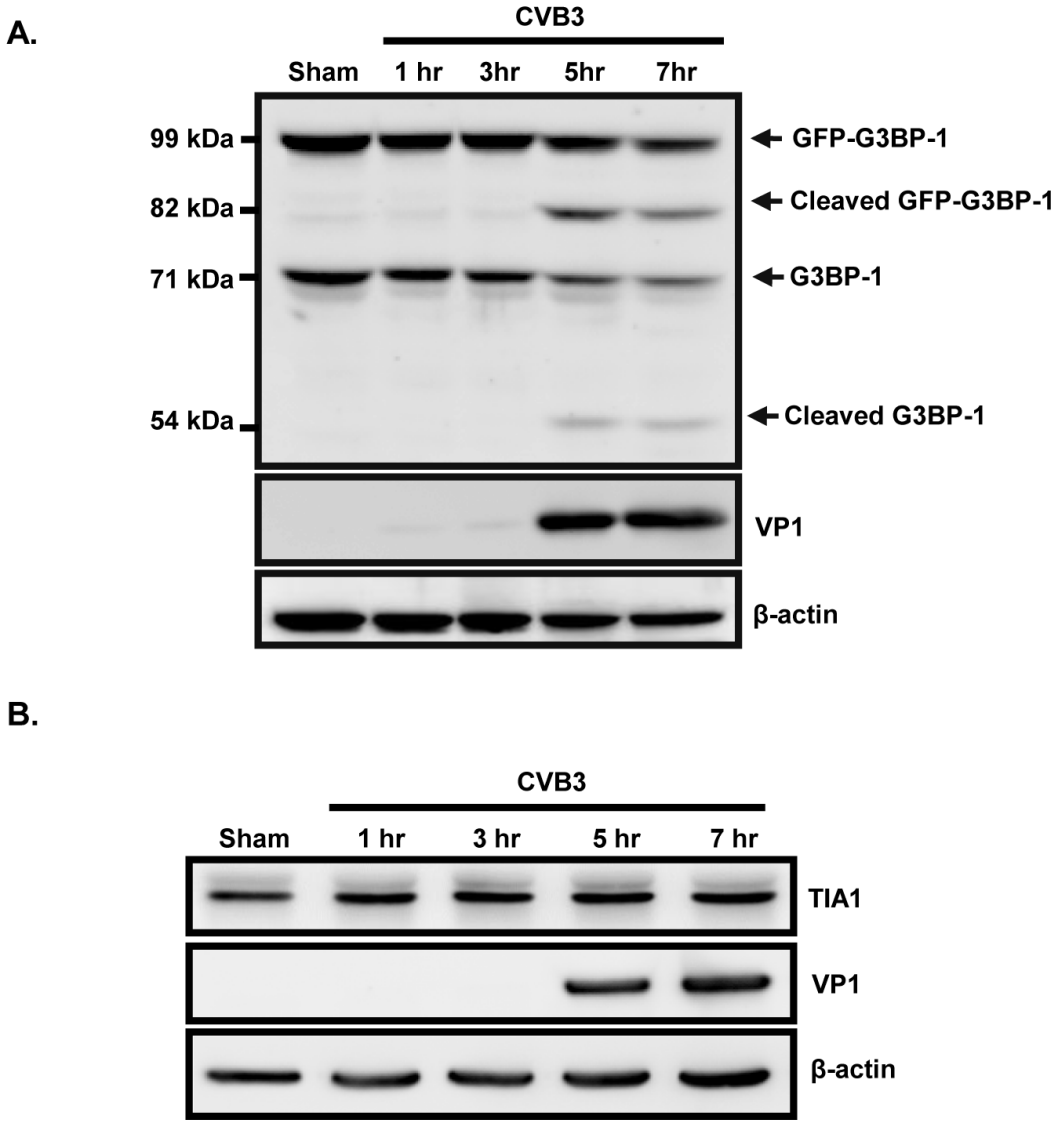
G3BP1 is cleaved at ∼5 hrs post-infection while TIA1 remains unchanged during the course of CVB3 infection. (A) HeLa cells stably expressing exogenous GFP-G3BP1 or (B) regular HeLa cells were either sham- or CVB3-infected at an MOI of 10 for various time points as indicated. Western blotting was performed to assess protein expression of G3BP1 using anti-G3BP1 antibody (A) and TIA1 using anti-TIA1 antibody (B). VP1 expression was used as an infection control and β-actin level was examined as a loading control.

We next examined the protein expression of G3BP1 and TIA1 during CVB3 infection in HeLa cells stably expressing GFP-G3BP1. Western blot results showed the reduction in expression of full length G3BP1 protein (exogenous 99 kDa, endogenous 71 kDa) at 5 and 7 hrs pi, accompanied by the appearance of a second band with smaller molecular weight (exogenous 82 kDa, endogenous 54 kDa) (**Figure 4A**), suggesting that G3BP1 is cleaved at 5 hrs pi. TIA1 remained unchanged along the course of CVB3 infection (**Figure 4B**).

### G3BP1 Cleavage is Due to Viral Protease 3C

**Figure 5:**
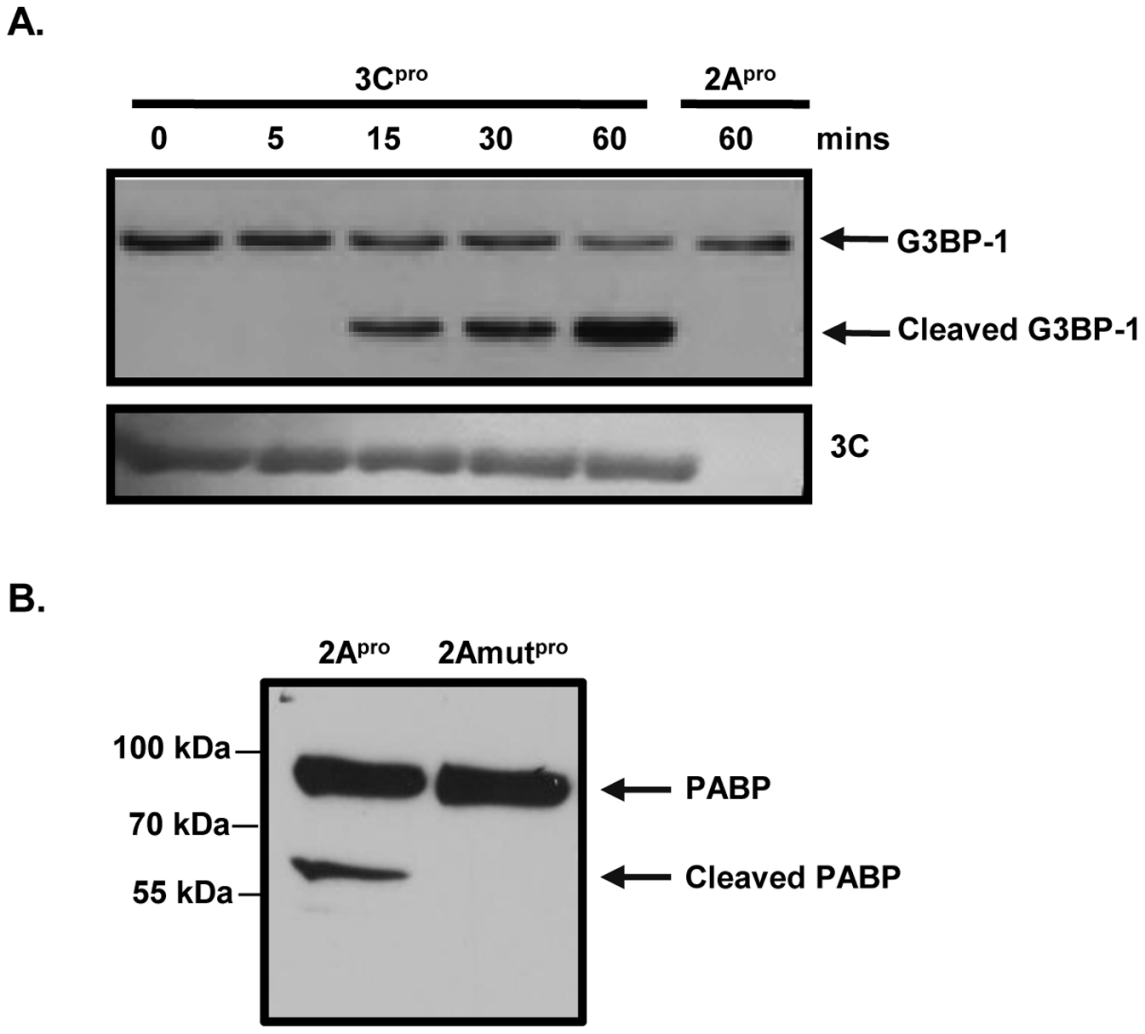
G3BP1 is cleaved by 3C^pro^. HeLa cell lysates were incubated with (A) purified 3C^pro^ for 0, 5, 15, 30, and 60 mins or 2A^pro^ for 60 mins, or (B) purified 2A^pro^ or 2Amut^pro^ for 60 mins. *In vitro* cleavage assay was performed as described in Materials and Methods and expression of G3BP1 and PABP was detected by western blotting.

To explore the mechanism of G3BP1 cleavage, viral proteases, 3C^pro^ and 2A^pro^, were purified and *in vitro* cleavage assays were conducted. **Figure 5A** showed that incubation with 3C^pro^ for increasing time points led to decreasing levels of full-length G3BP1 and increasing accumulation of G3BP1 cleavage fragments, suggesting that 3C^pro^ cleaves G3BP1. No cleavage bands were observed in 2A^pro^-treated cell lysates. To confirm the activity of purified 2A^pro^, we examined the cleavage of PABP, a known target of 2A^pro^ [2]. **Figure 5B** demonstrated that incubation with 2A^pro^, but not a catalytically inactive mutant 2A^pro^ (2Amut^pro^), resulted in the cleavage of PABP. Furthermore, we found that treatment of HeLa cells with Z-VAD-FMK, a pan-caspase inhibitor, had no effect on CVB3-induced G3BP1 cleavage (data not shown). Taken together, our results indicate that 3C^pro^ alone is sufficient to induce G3BP1 cleavage during CVB3 infection.

### G3BP1 is Cleaved at Amino Acid Q325 and a Cleavage Resistant Mutant Restores SG Formation

**Figure 6:**
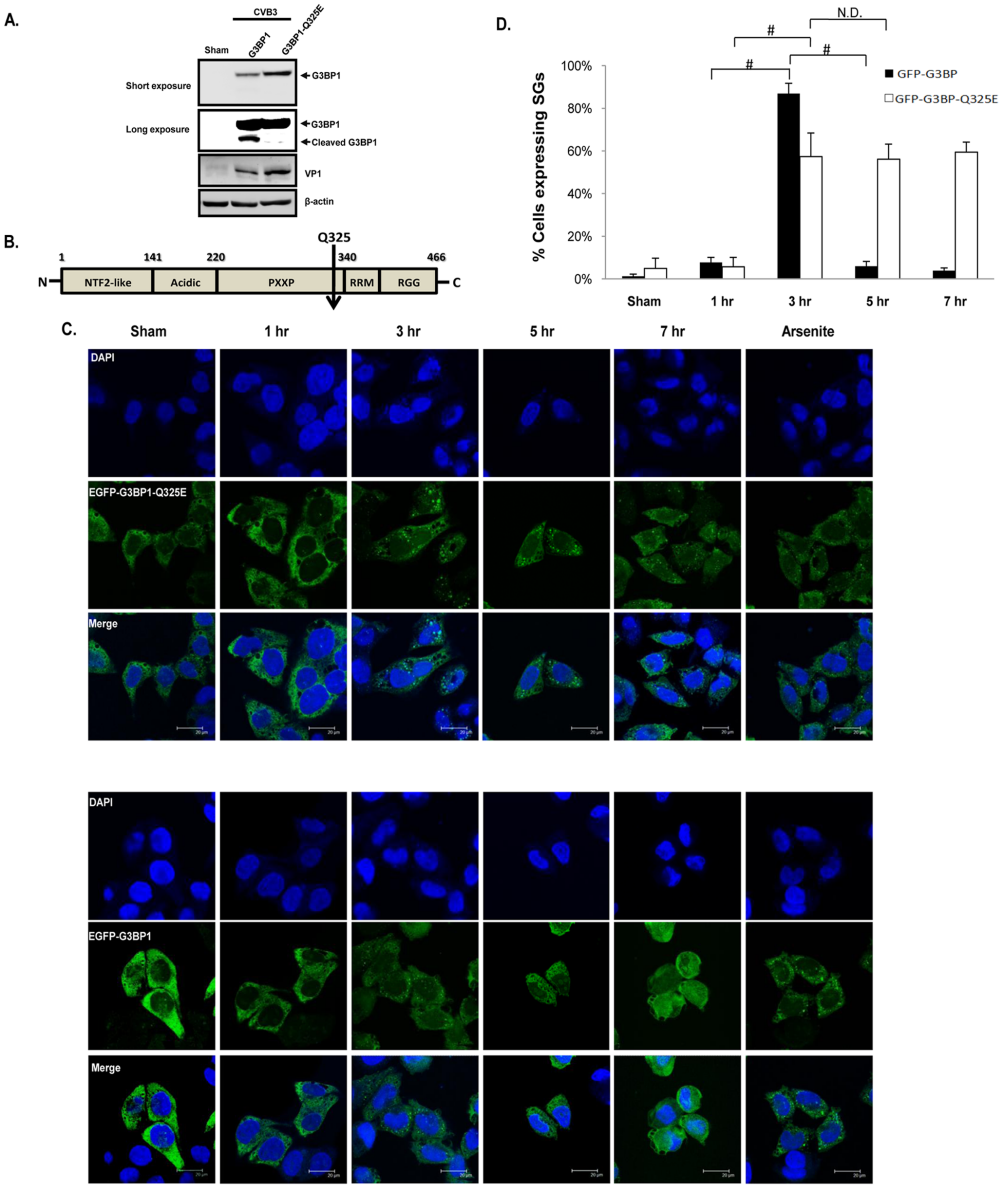
G3BP1 is cleaved at amino acid Q325 and a cleavage-resistant G3BP1 mutant restores SG formation at ∼5 hrs post-infection. (A) HeLa cells were transfected with FLAG-G3BP1 or FLAG-G3BP1^Q325E^ mutant for 48 hrs, followed by CVB3 infection at an MOI of 10 for 7 hrs. Western blotting was performed to examine G3BP1 cleavage using an anti-FLAG antibody. Protein expression of VP1 and β-actin was examined as an infection and loading control, respectively. (B) Schematic diagram of full length G3BP1 and the cleavage site. The arrow indicates the cleavage point at amino acid Q325 of G3BP1. NTF2-like, Nuclear Transport Factor 2-like; PXXP, SH3-domain binding domain of Ras-GAP; RRM, RNA Recognition Motif; RGG, Arginine-Glycine-rich region. (C) HeLa cells stably expressing GFP-G3BP1^Q325E^ (upper panels) or GFP-G3BP1 (lower panels) were sham- or CVB3-infected at an MOI of 10 for different times as indicated. Intracellular distribution of G3BP1 was examined using confocal microscopy. Cell nuclei were counterstained with DAPI. Cells treated with 50 mM arsenite for 1 hr were used as positive controls. (D) Quantitation of G3BP1-SG formation from (C). Results are presented as mean ± SD (n = 10 images), #p<0.001, N.D., No statistical difference.

To identify the cleavage site of G3BP1, we tested whether G3BP1 is also cleaved at amino acid 325, a previously identified cleavage site by PV 3C^pro^ [24]. We used a mutant G3BP1 with an amino acid mutation (Q325E). Western blot results demonstrated the absence of the cleavage product in FLAG-G3BP1^Q325E^-transfected HeLa cells following CVB3 infection, indicating that G3BP1^Q325E^ is cleavage resistant (**Figure 6A**). The cleavage of G3BP1 at Q325 separates its nuclear transport factor 2-like (NTF2-like) domain from the RNA recognition motif (RRM), which may lead to the disruption of the ability of G3BP1 to bind RNA and protein in order to induce SG formation (**Figure 6B**). Moreover, we conducted immunocytochemistry to determine whether non-cleavable G3BP1 (G3BP1^Q325E^) is able to restore SG formation after CVB3 infection. Results shown in **Figure 6C and D** demonstrated that SG assembly in cells stably expressing GFP-G3BP1^Q325E^ mutant was rescued at 5 hrs and 7 hrs pi. Live-cell fluorescent imaging using GFP-G3BP1^Q325E^ stable cell lines showed similar results (**Figure S2**). Together, our results suggest that cleavage of G3BP1 at amino acid Q325 contributes to G3BP1-SG disassembly observed during late stage of CVB3 infection.

### G3BP1 Negatively Regulates CVB3 Replication

**Figure 7:**
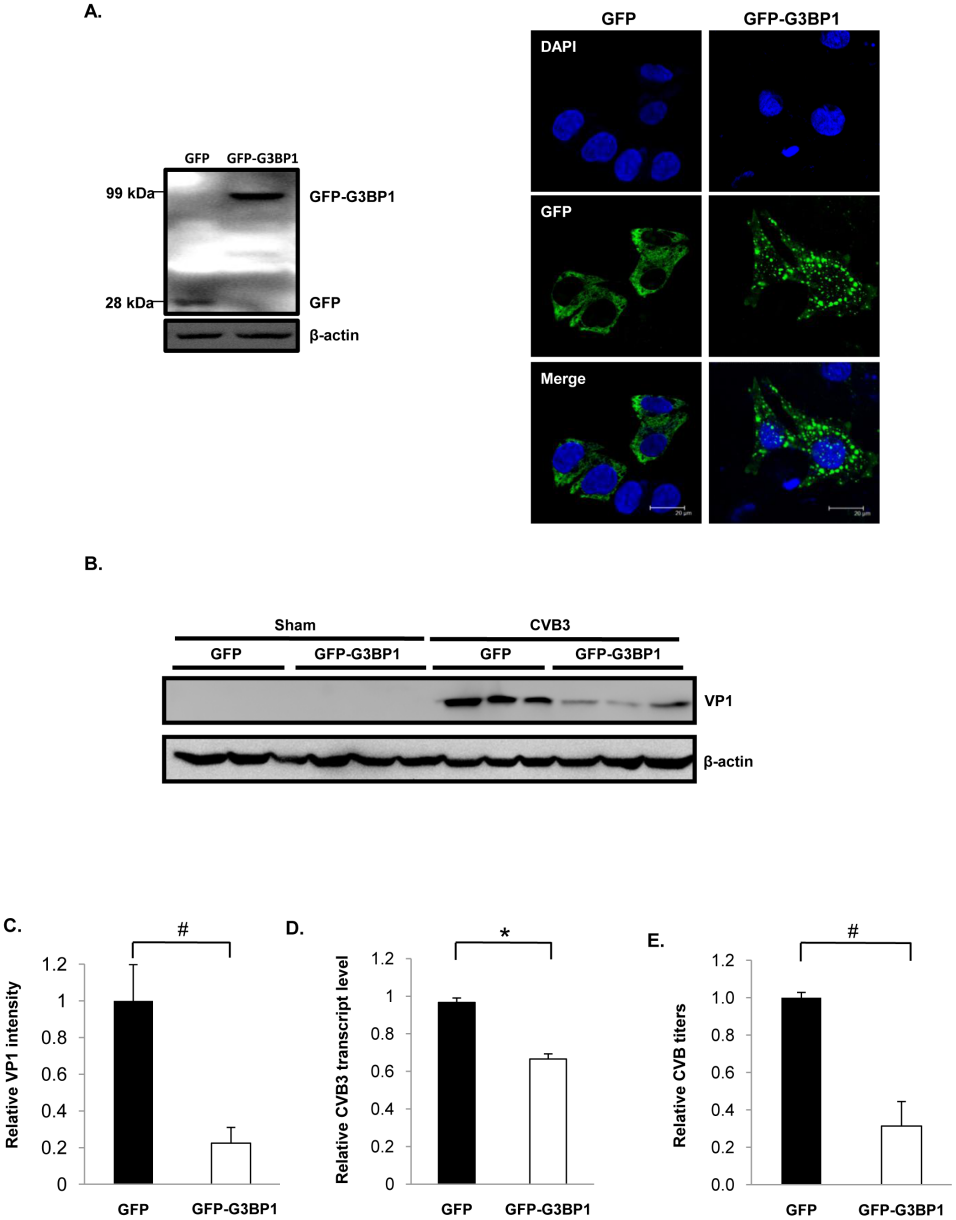
Overexpression of G3BP1 inhibits CVB3 replication. HeLa cells were transiently transfected with pEGFP-G3BP1 for 24 hrs, followed by CVB3 infection at an MOI of 1 for 16 hrs. (A) Western blotting (left) and confocal microscopy (right) were performed to examine protein level and intracellular distribution of GFP-G3BP1. (B) Western Blotting was performed to assess protein expression of VP1 and β-actin after CVB3 infection. (C) Densitometric analysis using ImageJ was performed on VP1 intensities relative to β-actin intensities of three independent experiments from (B). The value of sham was arbitrarily set as 1. (D) Quantitative RT-PCR was performed to examine viral transcript levels using primers specific to the IRES promoter of the CVB3 RNA transcript. Data is presented as transcript copy number relative to its empty vector control. The value of the empty vector control was arbitrarily set as 1. (E) Plaque assay was performed to examine the effect of G3BP1 overexpression on viral replication and results are presented as relative PFU/ml. The value of the empty vector control was arbitrarily set as 1. Results are presented as mean ± SD (n = 3), #p<0.001, *p<0.01.

**Figure 8:**
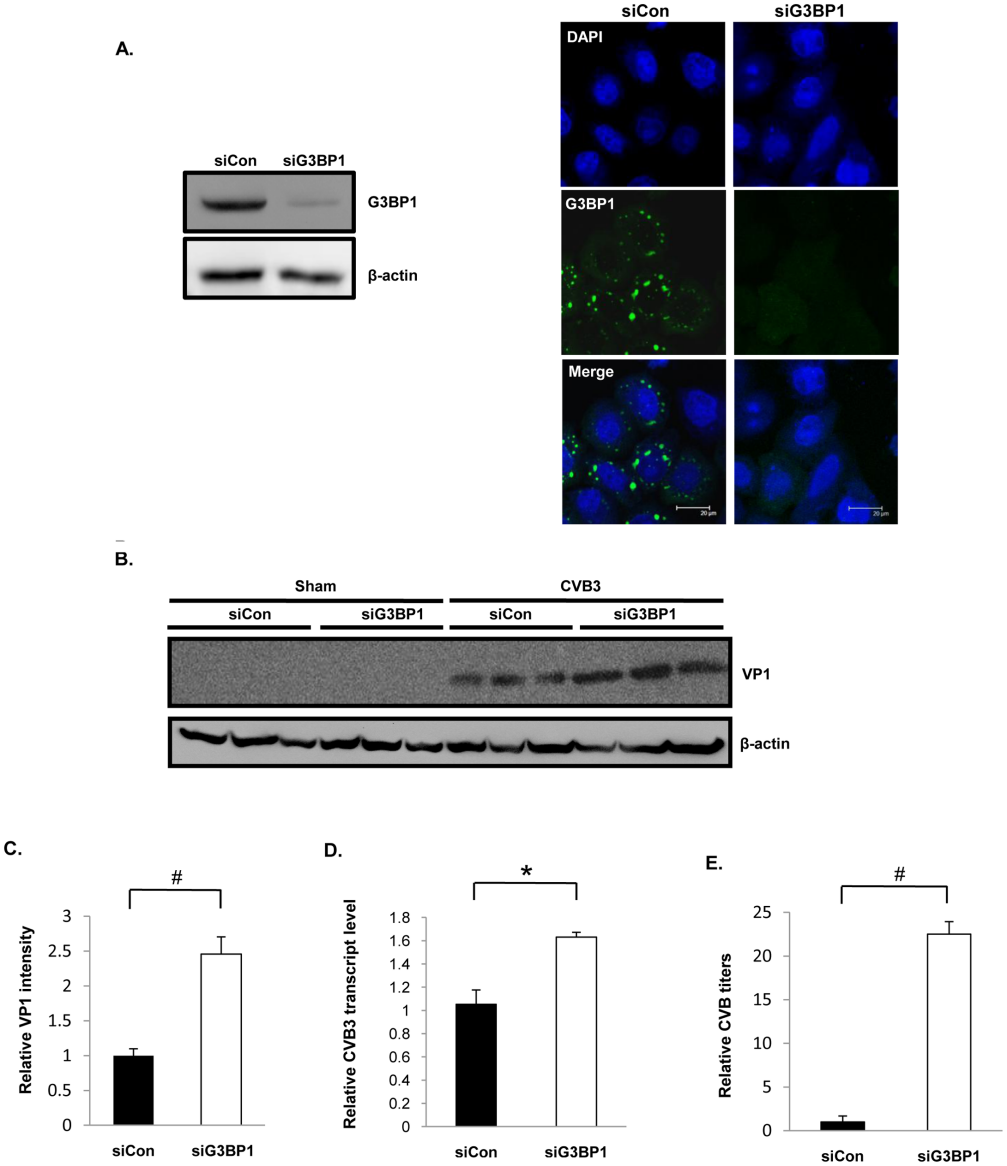
Knockdown of G3BP1 enhances CVB3 replication. HeLa cells were transfected with either control siRNA (siCon) or G3BP1-targeted siRNA (siG3BP1), followed by sham- or CVB3-infection at an MOI of 1 for 16 hrs. (A) Western blotting (left) and confocal microscopy (right) were performed to assess protein expression and intracellular distribution of GFP-G3BP1. (B) Western blotting was performed to examine VP1 and β-actin protein expression. (C) Densitometric analysis was performed on VP1 intensities of three independent experiments from (B) as described above. (D) Quantitative RT-PCR was performed to examine viral transcript levels as described above. Data is presented as transcript copy number relative to its empty vector control. The value of the siCon control was arbitrarily set as 1. (E) Plaque assay was performed to assess the effect of G3BP1 knockdown on viral replication and results are presented as relative PFU/ml. The value of the siCon control was arbitrarily set as 1. Results are presented as mean ± SD (n = 3), #p<0.001, *p<0.01.

To determine the significance of SGs in the course of CVB3 infection, G3BP1 was either overexpressed or knocked down in HeLa cells. We showed that overexpression of GFP-G3BP1 increased SG formation (**Figure 7A**), whereas knockdown of G3BP1 by siRNA led to the disappearance of G3BP1-SGs induced by CVB3 infection (**Figure 8A**). We further demonstrated that overexpression of GFP-G3BP1 resulted in marked decreases in VP1 protein expression (∼5 fold) (**Figure 7B and C**), viral transcripts (∼0.4 fold) (**Figure 7D**), and viral titers (∼3 fold) (**Figure 7E**). In contrast, knockdown of G3BP1 resulted in significant increases in VP1 protein expression (∼2.5 fold) (**Figure 8B and C**), viral transcripts (∼0.6 fold) (**Figure 8D**), and viral titers (∼22 fold) (**Figure 8E**). Our data suggests that G3BP1-SGs negatively regulate CVB3 replication.

### The C-terminal Cleavage Fragment of G3BP1 Inhibits G3BP1-SG Formation and Enhances CVB3 Replication

**Figure 9:**
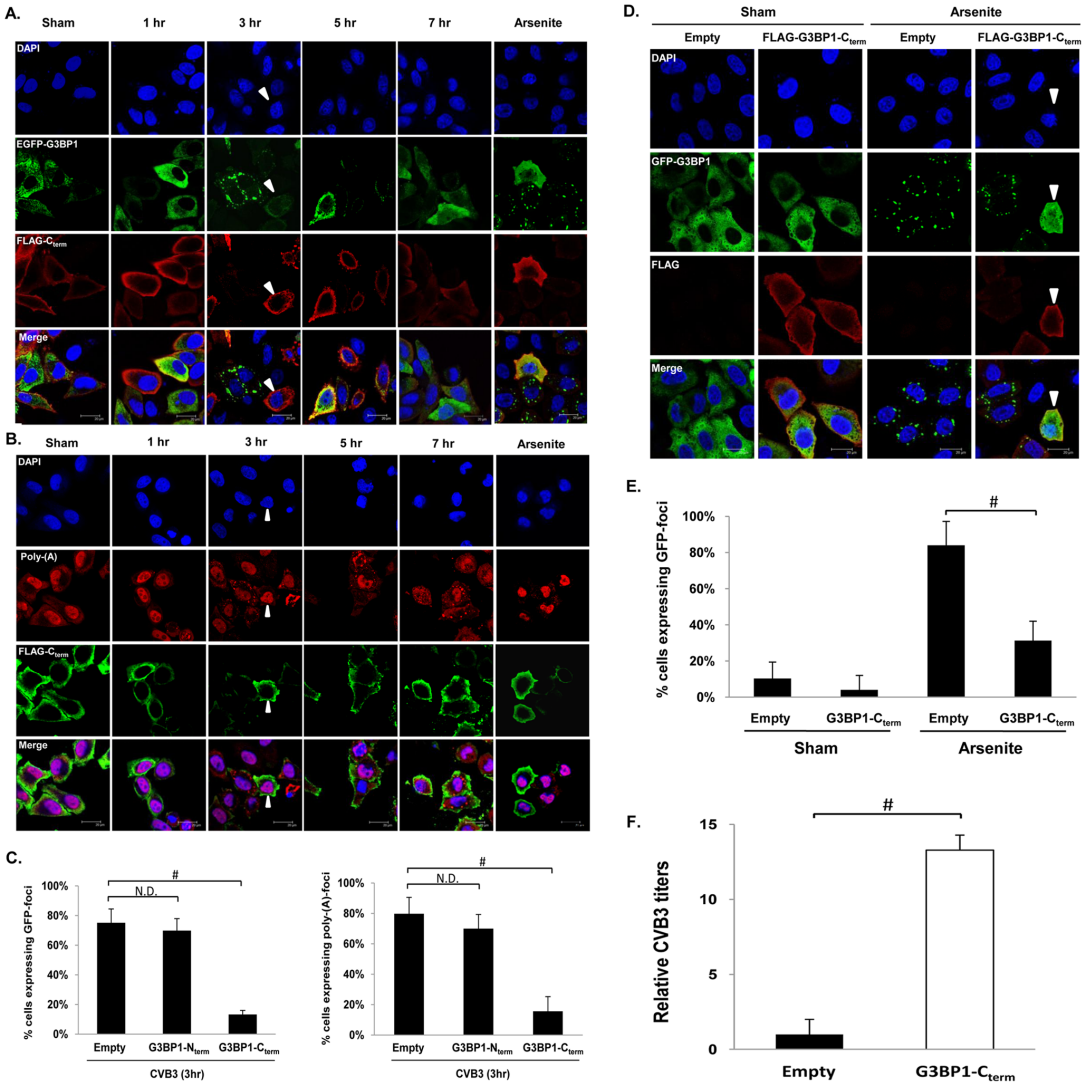
Figure 9. G3BP1-C_term_ fragment reduces SG formation and enhances CVB3 replication. (A, B) HeLa cells were co-transfected with pEGFP-G3BP1 and either FLAG-G3BP1-N_term_ (see Figure S3) or FLAG-G3BP1-C_term_ for 48 hrs, followed by sham- or CVB3-infection at an MOI of 10 for the indicated time points. G3BP1-C_term_ and G3BP1-N_term_ were stained using an anti-FLAG antibody. Arrow heads indicate cells expressing high levels of G3BP1-C_term_ but lacking distinct G3BP1(+)-SGs or poly-(A) granules. Cells treated with arsenite (50 mM) for 1 hr were used as positive controls. (C) Quantitation of GFP-G3BP1 foci and poly-A granules from (A and S3A) and (B and S3B) at 3 hr pi, respectively. Quantification was performed by counting cells expressing, empty vector, GFP-G3BP1-SGs or poly-A granules together with FLAG, and dividing by the total number of cells expressing FLAG. Results are presented as mean ± SD (n = 5 images), #p<0.001. N.D., No statistical difference. (D) HeLa cells were co-transfected with pEGFP-G3BP1 and FLAG-G3BP1-C_term_ for 48 hrs, and then treated with 50 mM arsenite for 1 hr. Immunostaining was conducted and representative images are displayed. (E) Quantitation of GFP-G3BP1 SGs from (D). The data is presented as mean ± SD (n = 5 images), #p<0.001. (F) Plaque assay was performed to assess the effect of G3BP1-C_term_ expression on CVB3 replication. Results are presented as relative PFU/ml. The value of the empty vector control was arbitrarily set as 1. The data is presented as mean ± SD (n = 3), #p<0.001.

We next explored whether the cleavage fragments of G3BP1 has any effects on SG formation. Confocal microscopy analysis showed that cells co-expressing G3BP1-N_term_ and GFP-G3BP1 displayed similar kinetics of G3BP1-SG assembly and disassembly as previously observed in wild-type G3BP1 (**Figures S3A and 9C**). Moreover, poly(A)-RNA granules assembled at 3 hrs pi and persisted throughout infection in G3BP1-N_term_-overexpressing cells (**Figures S3B and 9D**) as observed in **Figure 1C**. In contrast, cells expressing both G3BP1-C_term_ and GFP-G3BP1 displayed no or much smaller GFP punctates (arrow head) (**Figure 9A and C**), suggesting that the C-terminal fragment of G3BP1 negatively regulates SG formation. Similarly, the number of G3BP1-C_term_ expressing cells with poly(A)-granules at 3 hrs pi was significantly less as compared to control (**Figure 9B and C**). We also examined the influences of the C-terminal fragment of G3BP1 on arsenite-induced SG formation. **Figure 9D** showed that HeLa cells expressing G3BP1-C_term_ inhibited arsenite-induced SGs, suggesting that the inhibitory effect of G3BP1-C_term_ on SG formation is not CVB3-specific.

Finally, we examined the impacts of the C-terminal portion of G3BP1 on viral replication. **Figure 9E** showed that overexpression of G3BP1-C_term_ resulted in significant increases in virus titers as compared to empty control. Taken together, our results suggest that the C-terminal fragment of G3BP1 acts as a dominant-negative inhibitor for G3BP1-SG formation and positively affects viral growth.

### G3BP1-SGs do not Co-localize with CVB3 Sense-strand

**Figure 10:**
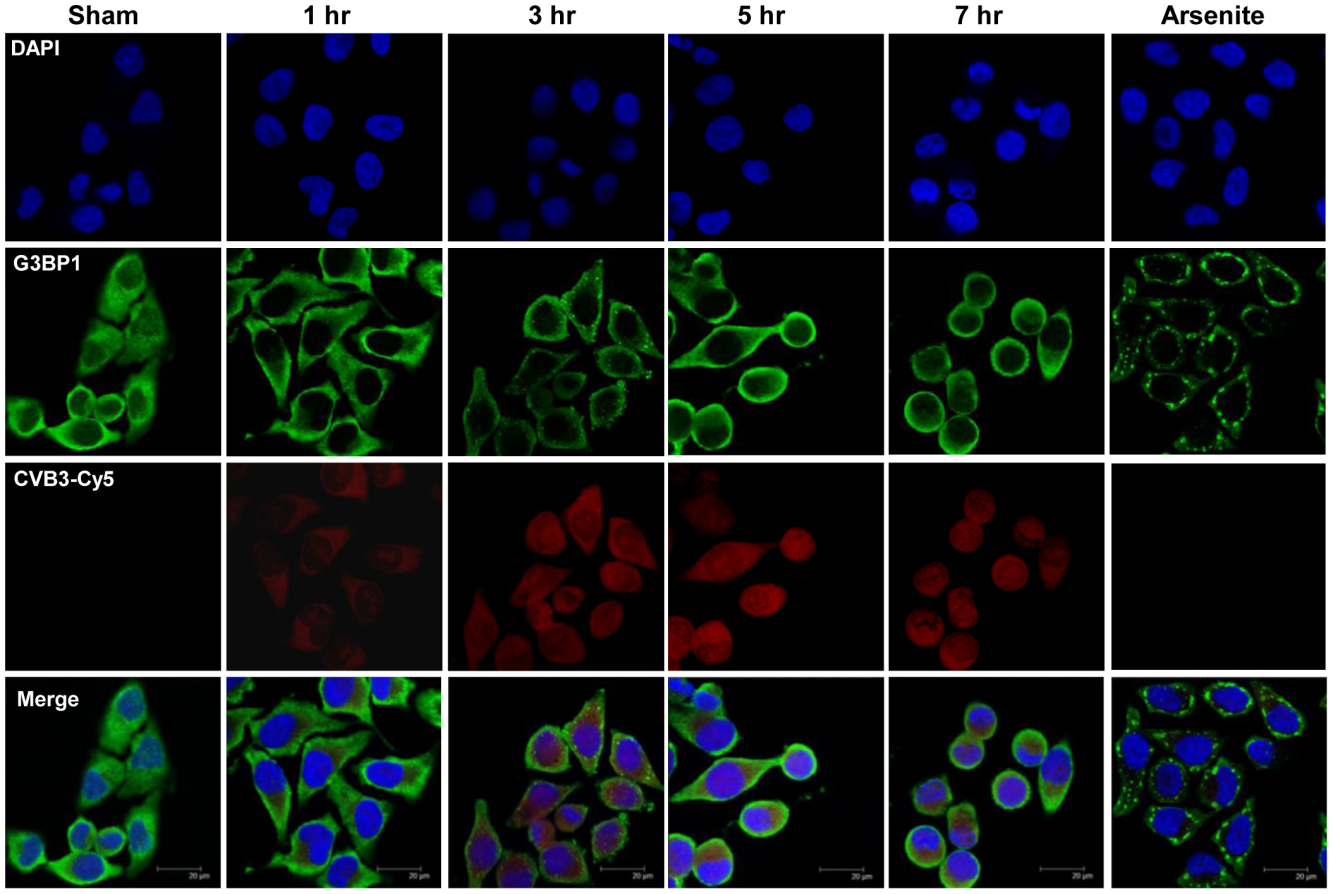
G3BP1-SGs do not localize with CVB3 sense-strand. HeLa cells were infected with CVB3 at an MOI of 10 for the indicated time points, followed by *in situ* hybridization and immunostating as described in Materials and Methods. Endogenous G3BP1 was detected using an Alexa-488 secondary antibody. CVB3 sense-strand was detected by the synthetically conjugated Cy5 fluorophore.

To elucidate whether SGs bind to viral transcripts to block the interaction of factors necessary for IRES translation, HeLa cells were sham- or CVB3-infected for the indicated time points, then probed with an oligonucleotide complimentary to the sense-strand of CVB3 transcripts, synthetically conjugated to a Cy-5 fluorescent tag (red), and immunostained for endogenous G3BP1 (green). The sense-strand of CVB3 transcripts was chosen because it is used for both replication as well as protein translation. Results shown in **Figure 10** demonstrated the early formation and late disassembly of G3BP1-SGs over the time course of CVB3 infection, which is in line with the findings in Figure 1A; however, red fluorescence signal was found to be homogenously distributed with no co-localization with G3BP1-SGs. Furthermore, this binding is specific to CVB3 sense-strand since no red fluorescence was observed in sham- or arsenite-treated cells, but gradually increased over the time course of CVB3 infection. Our findings that CVB3 sense-strand does not interact with G3BP1-SGs suggest that G3BP1-SGs may function to reduce CVB3 replication by other mechanisms.

## Discussion

Viruses are obligated to evolve molecular mechanisms to evade host antiviral responses as well as hijack host protein machinery for their own benefit. Many viruses have been documented to interact with host SGs [14]. In this study, we found that SGs are assembled early following CVB3 infection using multiple SG markers such as G3BP1, TIA1, HuR and Poly-A RNA via confocal microscopy, live-cell fluorescent imaging, and by both transmission- and immuno-electron microscopy. Furthermore, we demonstrated that SGs are actively disassembled at late times during infection where G3BP1, TIA1 and HuR dissociate from SGs back into the cytoplasm, while poly-A granules persist, suggesting that G3BP1-SGs are specifically targeted while other RNA granules such as P-bodies are not. In addition, we observed partial co-localization of G3BP1 and O-linked N-acetylglucosamine (data not shown), a sugar moiety modification recently reported to be localized to SGs [38], during CVB3 infection. Our findings indicate that CVB3-induced SGs may be functionally and compositionally different from those formed under other stresses. Interestingly, by TEM, cytosolic aggregates with ribosomal-like structures around the perimeter were observed adjacent to mitochondrial structures in CVB3-infected cells. Consistent with this finding, IEM showed G3BP1-positive staining for aggregates adjacent to mitochondria. Furthermore, we found that G3BP1-SGs are actively inhibited and disassembled late during CVB3 infection.

CVB3 infection causes shutoff of host protein translation as early as 1 hr after CVB3 infection [39]. Our data suggests that increased formation of SGs is likely due to active cleavage of eIF4G by 2A^pro^, which occurred early during viral infection, rather than the canonical eIF2α phosphorylation which took place at ∼5 hrs pi. We observed the cleavage of a primary SG marker protein G3BP1 after CVB3 infection and that the cleavage was independent of host caspase activity (data not shown) but was directly due to 3C^pro^ activity. Like other viruses in the *Picornaviridae* family, such as PV and EMCV [24], [27], G3BP1^Q325E^ was cleavage resistant. This cleavage separates the NTF2-like domain from the RRM domain of G3BP1, which may lead to the failure to form specific protein-RNA aggregates. We speculate that dissociation of NTF2-like domain from RRM may be a conserved mechanism across *Picornaviridae* viruses. We also demonstrated that overexpression of GFP-G3BP1 resulted in a reduction of viral protein expression, transcripts, and viral titers. In contrast, siRNA knockdown of endogenous G3BP1 led to an increase in VP1 expression, transcripts, and viral titers, indicating that SGs negatively regulate CVB3 replication. It is important to note that the ∼22.5 fold increase in viral titres after siG3BP1 treatment may be an additive effect of secreted interferon proteins that were collected in the supernatant of CVB3-infected cells.

Although G3BP1-SGs are disrupted during PV, EMCV and CVB3 infection, the pathological significance of the cleavage fragments remains unclear. Our studies showed that 3C^pro^ cleaved G3BP1 at Q325, producing a C-terminal fragment of G3BP1 that inhibits SG formation and promotes CVB3 replication, whereas the N-terminal fragment of G3BP1 failed to result in any difference in G3BP1-SG formation and disassembly. Future studies will be required to further explore the molecular mechanism by which G3BP1-C_term_ interferes with G3BP1-SG formation and the molecular basis of the proviral action of G3BP1-C_term_. It is important to note that the cleavage of full-length G3BP1 may not be the primary cause of SG-disassembly, but actually the production of a dominant-negative peptide. Thus complete cleavage of full length protein may not be entirely necessary.

Our data using a CVB3-Cy5 oligonucleotide showed that G3BP1-SGs and positive-sense viral transcripts failed to co-localize. Furthermore, CVB3 transcripts lack formation of cytosolic punctates, which may suggest that CVB3 transcripts do not necessarily require any distinct host organelles for replication and protein production. Therefore, G3BP1-SGs may cause activation of downstream effectors such as interferon and NF-kB pathways to negatively impact CVB3 replication.

Although the antiviral mechanisms were not addressed in the current study, based on our previous reports [26], [27], we hypothesize that G3BP1-SGs contribute to an interferon antiviral response to CVB3 infection. We have previously demonstrated that antiviral-SGs contain a dsRNA sensor protein RIG-I, which binds to IPS-1, a mitochondrial-membrane protein, resulting in activation of a type I interferon response [26]. In addition, we have recently shown that EMCV infection disrupts cytoplasmic G3BP1-SGs, resulting in a diminished type I interferon response [27]. Altogether, we believe that G3BP1-SGs function as an antiviral defence mechanism by interacting with mitochondrial surface proteins in response to foreign dsRNA and host protein translation inhibition.

CVB3 has been demonstrated to interfere with cellular translation, apoptosis/survival, and NF-kB activation through the action of its proteases [1], [3], [40]–[42]. The observation in this study of the dominant-negative effect of the G3BP1-C_term_ cleavage fragment on G3BP1-SG formation extends our understanding of how *Picornaviridae* viruses may strategically target proteins critical for cellular responses. This mechanism might serve as a second level of regulation to further reduce SG formation and promote viral replication. Future investigation is warranted to further investigate the antiviral effects of G3BP1-SGs and how the cleavage fragments may possibly be multifaceted in inhibiting antiviral responses.

## Supporting Information

Figure S1 **Stress Granules are induced upon CVB3 infection.** HeLa cells stably expressing GFP-G3BP1 were CVB3 infected at an MOI of 5. GFP-G3BP1 expression/localization was visualized using real-time fluorescent imaging over 460 mins while capturing images at 10 min intervals.
(AVI)

Figure S2 **Cleavage resistant GFP-G3BP1^Q325E^ restores stress granules in late stage of CVB3 infection.** HeLa cells stably expressing GFP-G3BP1^Q325E^ were CVB3 infected at an MOI of 5. GFP-G3BP1^Q325E^ expression/localization was visualized using real-time fluorescent imaging over 460 mins while capturing images at 10 min intervals.
(AVI)

Figure S3 **G3BP1-N_term_ does not alter G3BP1-SG formation and disassembly in CVB3 infection.** (A, B) HeLa cells were co-transfected with pEGFP-G3BP1 and FLAG-G3BP1-C_term_ for 48 hrs, followed by sham- or CVB3-infection at an MOI of 10 for the indicated time points. (A) G3BP1-N_term_ was stained using an anti-FLAG antibody. (B) Poly-(A)-mRNA was stained by *in-situ* hybridization. Cells treated with arsenite (50 mM) for 1 hr were used as positive controls.
(TIF)
