# Supplementary figures and images for: Production of a Dominant-Negative Fragment Due to G3BP1 Cleavage Contributes to the Disruption of Mitochondria-Associated Protective Stress Granules during CVB3 Infection
Source: PLoS One. 2013 Nov 18;8(11):e79546. doi: 10.1371/journal.pone.0079546 (PMC3832613; doi:10.1371/journal.pone.0079546)

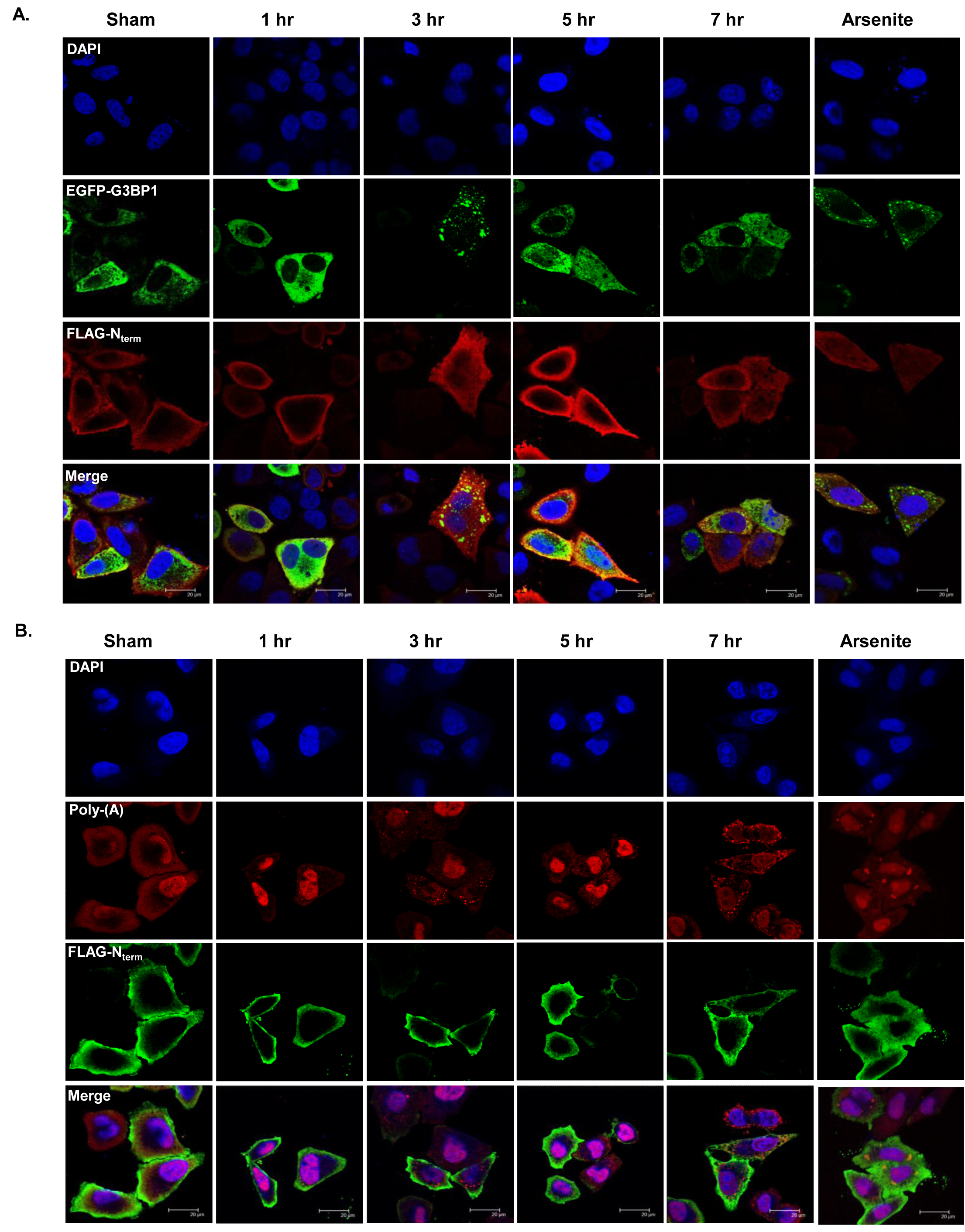

Supplement: Figure S3 — G3BP1-Nterm does not alter G3BP1-SG formation and disassembly in CVB3 infection. (A, B) HeLa cells were co-transfected with pEGFP-G3BP1 and FLAG-G3BP1-Cterm for 48 hrs, followed by sham- or CVB3-infection at an MOI of 10 for the indicated time points. (A) G3BP1-Nterm was stained using an anti-FLAG antibody. (B) Poly-(A)-mRNA was stained by in-situ hybridization. Cells treated with arsenite (50 mM) for 1 hr were used as positive controls. (TIF) [file pone.0079546.s003.tif]
